# Overexpression of the Promigratory and Prometastatic PTK7 Receptor Is Associated with an Adverse Clinical Outcome in Colorectal Cancer

**DOI:** 10.1371/journal.pone.0123768

**Published:** 2015-05-11

**Authors:** Anne-Catherine Lhoumeau, Sébastien Martinez, Jean-Marie Boher, Geneviève Monges, Rémy Castellano, Armelle Goubard, Marie Doremus, Flora Poizat, Bernard Lelong, Cécile de Chaisemartin, Florence Bardin, Patrice Viens, Jean-Luc Raoul, Thomas Prebet, Michel Aurrand-Lions, Jean-Paul Borg, Anthony Gonçalves

**Affiliations:** 1 CRCM, Team Cell Polarity, Cell signalling and Cancer “Equipe labellisée Ligue Contre le Cancer”, Inserm, U1068, Marseille, F-13009, France; 2 Institut Paoli-Calmettes, Marseille, F-13009, France; 3 Aix-Marseille Université, Marseille, F-13284, France; 4 CNRS, UMR7258, F-13009, Marseille, France; 5 Department of Biostatistics, Institut Paoli-Calmettes, Marseille, France; 6 Department of Biopathology, Institut Paoli-Calmettes, Marseille, France; 7 Department of Surgical Oncology, Institut Paoli-Calmettes, Marseille, France; 8 Department of Medical Oncology, Institut Paoli-Calmettes, Marseille, France; 9 Department of Hematology, Institut Paoli-Calmettes, Marseille, France; Queen Mary Hospital, HONG KONG

## Abstract

Biomarkers and novel therapeutic targets are urgently needed in colorectal cancer (CRC). The pseudo tyrosine kinase receptor 7 (PTK7) is involved in planar cell polarity and it is deregulated in various malignancies, including CRC. Yet, little is known about its protein expression in human CRC, or about a possible correlation of its expression with clinical endpoints. Using a clinically annotated Tissue MicroArray (TMA) produced from from 192 consecutive CRC patients treated by initial surgery, we examined PTK7 expression by immunohistochemistry in tumoral tissue and matched normal mucosae, and correlated its expression with clinico-pathological features and patient outcome. PTK7 depletion by specific shRNA in HCT116 and HCT15 CRC cell lines was found to affect cell proliferation, resistance to drugs and cell migration. Tumor growth and metastatic phenotype were investigated *in vivo* using a xenograft mouse model of CRC cells with modulated expression of PTK7 levels. PTK7 was significantly up-regulated in CRC tissue as compared to matched healthy mucosae, and significant overexpression was found in 34% of patients. PTK7 overexpression was significantly associated with a reduced metastasis-free survival in non-metastatic patients. In HCT116 and HCT15 cells, shRNA PTK7 reduced migration but did not affect cell proliferation and resistance to drugs. In a xenograft mouse of HCT15 cells, downregulation of PTK7 led to reduced tumor growth, whereas its overexpression in PTK7-negative cancer cells led to increased metastatic events. PTK7 expression thus represents a potential prognostic biomarker and a novel therapeutic target in CRC.

## Introduction

With 447 000 cases and 215 000 deaths per year in Europe, colorectal cancer (CRC) remains a major public health issue [1,2]. Integration of 5-FU- and oxaliplatin-based adjuvant chemotherapy to surgical resection in node positive-patients has improved survival [3,4], but a significant number of these patients still ultimately relapse and die from metastatic disease. In the same time, node-negative patients are usually not treated with adjuvant systemic treatment, whereas some of them could benefit from this strategy [5]. Thus, identification of valid and robust biomarkers that may distinguish a group of patients presenting significant risk of recurrence is urgently needed. In addition, even though some molecular targeted therapeutics have contributed to increase survival in metastatic CRC [6–9], none of them was demonstrated to improve survival in the adjuvant setting [10,11]. Therefore, it is still eagerly necessary to identify molecular actors that play a relevant role in colon cancer biology and may serve as targets for novel biological therapies.

The cell surface receptor PTK7, also known as colon carcinoma kinase-4 (CCK-4), is an evolutionary conserved member of the receptor tyrosine kinase superfamily, which was first identified in human normal melanocytes [12] and in human colon carcinoma [13]. Composed of seven extracellular immunoglobulin domains, a transmembrane region and an intracellular tyrosine kinase domain, it has a defective kinase activity and no known ligand. Although its exact biological role is unclear, recent evidence has linked PTK7 to the planar cell polarity (PCP) pathway [14]. While the apico-basal polarity organizes epithelial cell attachment along an x-y axis, PCP controls the position of cells within the plane of an epithelial structure, guaranteeing that they are oriented in the same direction, and thereby plays a major role in various developmental processes, including epithelial cell differentiation and movements [15]. Of note, deregulation of PCP can cause various pathological disorders, including cancer. Well-known regulators of PCP include Wnt ligands and Frizzled (Fz) receptors, which activate the Dishevelled adaptor at the plasma membrane, and initiate the so-called Wnt pathway, either in its canonical (β-catenin-dependent) or non-canonical (β-catenin-independent) organization [16]. PTK7 was suggested to regulate PCP since its mutation in Xenopus or in the mouse led to obvious PCP-related developmental disorders, including neural tube closure defects [17,18]. In addition, PTK7 was shown to directly interact with Dishevelled, leading to its recruitment at the membrane and subsequent phosphorylation/activation by Fz7 [19]. We and others have demonstrated that PTK7 can also activate the canonical Wnt pathway in a kinase domain-dependent manner [18,20].

Recently, PTK7 was found to be overexpressed in various human cancers, including epithelial tumors such as gastric, breast, esophagus, biliary duct and lung cancers [21–26] but also in sarcoma [27] and in hematological malignancies, including acute and chronic myeloid leukemias [28–30]. Surprisingly, whereas PTK7 was first described in human colon cancer cell lines [13], no data are currently regarding its protein expression in CRC tissues from clinically annotated patients. Thus, we have evaluated PTK7 protein expression on a TMA composed of CRC primary tissues and matched healthy mucosae, and assessed possible correlations of its expression with conventional clinical and pathological parameters, as well as with patient outcome. PTK7 expression was thus shown to be associated with metastatic outcome and reduced survival in non-metastatic CRC patients. We then investigated the impact of modulating its expression in preclinical models and found that PTK7 induces a pro-migratory and pro-metastatic phenotype, consistent with the clinical data.

## Materials and Methods

### Ethic statement

The study was approved by the Institut Paoli-Calmettes (IPC) Institutional Review Board (IRB, Comité d’Orientation Stratégique, COS). The IRB did not consider as mandatory to obtain informed consent from patients, but data were analyzed after all patient information have been fully anonymized. For animal research, all experiments were performed in agreement with the French Guidelines for animal handling and approved by the Ethics Committee in Animal Experimentation of Marseille (C2EA-14). The animals were housed in Specific Pathogen Free conditions, in individually ventilated cages (Tecniplast, France, Sealsafe Plus Mouse—Mouse IVC Green Line) with 4–5 companions.

All mice were allowed free access to autoclaved and filtered water and irradiated food in a 10–14-hour light/dark cycle, with room temperature at 21±2°C and humidity at 55±15%. All cages contained wood shavings, bedding and a poplar wood wool nest (Anibed, France) for environmental enrichment.

During the postoperative period, pain was relieved by a subcutaneous administration of meloxicam (Metacam injectable, Boehringer Ingelheim, 1mg/kg once daily for 3 days) and Baytril (Bayer)-supplemented water was additionally administered to mice for 7 days prior xenograft for the prevention of infection during 3 weeks following surgery.

Humane killing of mice was performed using inhaled carbon dioxide anaesthesia in accordance with the Ethics Committee in Animal Experimentation of Marseille (C2EA-14)

### Patients and tissues

Archival tissues from 192 consecutive patients with stages I to IV colorectal carcinoma (CRC) treated at our institution (Institut Paoli-Calmettes, Marseille, France) by initial surgical resection were collected between 1990 and 1998. Staging used the American Joint Committee on Cancer criteria [31]. All samples were formalin fixed and paraffin embedded, and evaluated in the Biopathology department. Patients received adjuvant 5FU-based chemotherapy in case of lymph node involvement or metastatic disease according to standard recommendations. After treatment completion, surveillance was performed at 3-month intervals for the first 2 years and at 6-month intervals thereafter. Patients were monitored for metastatic relapse by clinical exam and blood tests, yearly chest X-ray and liver ultrasound and/or CT scan. Clinical and pathological features as well as outcome characteristics were extracted from our prospectively maintained institutional database. In addition, to investigate possible differences in PTK7 expression between metastatic and primary tissues, 7 PTK7-positive liver metastases and paired primary CRC were selected for comparative immunohistochemistry.

### Tissue microarrays construction

TMA were prepared as previously described [32,33]. For each sample, 3 representative areas were selected from a haematoxylin–eosin-stained section of a donor block. Core cylinders with a diameter of 0.6 mm were punched and put on 3 separate recipient paraffin blocks using a specific arraying device (Beecher Instruments, Silver Spring, MD, USA). The recipient block contained pairs of tumor and normal mucosa, as well as control normal and benign tissues (small intestine, adenomas) and cell line pellets. Five μm sections of the resulting TMA block were used for IHC analysis after transfer onto glass slides. Two colon cancer cell lines (CaCo2, HT29) and one gastric cancer cell line (HGT1) were used as controls.

### Immunohistochemistry analysis

For PTK7 staining goat IgG, anti-human/mouse/rat PTK7/CCK-4 affinity-purified polyclonal antibody was obtained from R&D Systems (Minneapolis, MN, USA). Deparaffinisation was performed in histolemon (Carlo Erba Reagenti, Rodano, Italy) and sections were rehydrated in graded alcohol. For antigen enhancement, sections were first incubated in target retrieval solution (Dako, Glostrup, Denmark). Staining reactions were performed at room temperature with an automatic stainer (Dako Autostainer) and were conducted as follows: after washes in appropriate phosphate buffer, followed by quenching of endogenous peroxidase activity with 3% H2O2, slides were first blocked with serum (Dako) during 30 min and then incubated with the above described PTK7 antibody for 1 h (dilution 1/200). After washes, slides were incubated with biotinylated antibody against goat immunoglobulin (dilution 1/200; Dako) for 25 min and then by streptavidin-conjugated peroxydase (Dako REALkit). Diaminobenzidine was used as the chromogen. Haematoxylin was used for counterstaining, and coverslips were mounted using Curemount (Instrumedics, Hackensack, USA) solution. They were examined under a light microscope by two pathologists (GM, FP). The following parameters were addressed: staining intensity (0: absent, 1: low, 2: medium, 3: high), percentage of stained cells, and staining features (nucleus, membrane or cytoplasmic staining).

For KI67 staining, mouse IgG, anti-Human Ki67 Clone MIB-1 from Dako was used as previously described [34].

### Cell lines and cell culture

The CRC cell lines, HCT116 and HCT15, were provided by the ATCC. HCT116 and HCT15 cells were maintained in Dulbecco's Modified Eagle Medium supplemented with 10% fetal bovine serum and 1% penicillin-streptomycin.

### Culture of L cells, Wnt5a/ Wnt3a production, and B16F10 in vivo metastasis assays

See S1 Materials and Methods.

### Design of specific shRNA and production of viral particles

shRNA sequences specific for PTK7 constructs were designed as described in S1 Materials and Methods. These oligonucleotides were purchased from Invitrogen, France and cloned into pLKO.1-GFP vector using AgeI and EcoRI restriction site. A scramble non-targeting shRNA was used as control.

Viral particles containing shRNA were produced by transient transfection of HEK 293T cells with packaging system vectors (PSPAX: plasmid 12260 and VSVG: plasmid 12259, Addgene, Cambridge, USA) and pLKO.1-shRNAs-GFP. Viral supernatants were harvested 48h after transfection, filtered and used to infect HCT 116 and HCT15 cells in presence of 4μg/ml of Polybrene. Infection efficiency was controlled by GFP expression in infected cells. Cells were sorted by flow cytometry using GFP expression (BD Aria2, Becton Dickinson, San Jose, CA, USA) and shRNA efficiency was controlled by Western Blot and qRT-PCR analysis.

### Protein extraction and immunobloting

Cells were lysed in lysis buffer (50mM Hepes, 150mM NaCl, 1mM EDTA, 1mM EGTA, 10% glycerol, 1% Triton X-100, 25mM NaF, 10μM ZnCl2) supplemented with 0,5mM phenylmethylsulfonyl fuoride (PMSF), 1mM orthovanadate, 1mM β-glycerophosphate and a protease inhibitor cocktail (Sigma-Aldrich, USA). Lysates were centrifuged at 13000 rpm for 10 min at 4°C. Pellets were discarded and protein concentration was adjusted using Bradford assay (BioRad). Proteins were resolved by SDS-PAGE, transferred to nitrocellulose filters, blocked 1h at room temperature in Tris-Buffered Saline / 5% non-fat dry milk / 0,1% Tween20, and blotted overnight with primary antibodies in blocking solution (rat monoclonal anti-PTK7 generated in the laboratory; mouse monoclonal αTubulin antibody, Sigma-Aldrich, USA). After extensive washings in TBS / 0,1% Tween20, filters were incubated 1h at room temperature (RT) with a HRP-conjugated secondary antibody before being revealed with an enhanced chemiluminescence substrate (West Pico, Thermo Scientific, USA). Acquisition was performed with a G-BOX imager (Ozyme, France).

### Quantitative RT-PCR analysis

Total RNA was isolated using RNeasy mini kit (Qyagen, Netherlands). The RNA pellets were air-dried and dissolved in ultrapure water. cDNA was generated with High Capacity cDNA Reverse Transcription Kit (Applied Bio-systems, USA) following manufacturer instructions. SYBR Premix was prepared with 10-pM primers and 1μL cDNA template. qRT-PCR on 96-well optical plates was performed using the above reagents, and the products were analyzed on an ABI Prism 7500 (Applied Bio-systems, USA). The level of expression of PTK7 was normalized with GAPDH and represented as a relative expression. Probes were as follows: PTK7 fwd 5′- CAGTTCCTGAGG ATTTCCAAGAG -3 and rev 5′-TGCATAGGGCCA CCT TC-3′; GAPDH fwd 5’-ATGGGGAAGGTGAAGGT-3’ and rev 5’-AAGCTTCCCGTTCTCAG-3’

### Cell proliferation assay

Cell proliferation was assessed by CellTiter 96 assay (Promega, USA). Cells were seeded at a density of 5000 cells in 96-well plates and incubated in DMEM containing 10% FCS for the indicated time. Then plates were incubated with CellTiter 96 at 37°C for 2h. Absorbance at 490nm was measured using a 96-well plate reader.

### Drug resistance assays

Cells were seeded at a density of 5 000 cells in 96-well plates and incubated in DMEM containing 10% FCS for the 24h. Then, drugs (Irinotecan, 5-Fluorouracil, and Cisplatin) were added and cells were further incubated for 72h CellTiter 96 was then added and plates were incubated at 37°C for 2h. Absorbance at 490nm was measured using a 96-well plate reader.

### Cell migration assay

Cells were seeded at a density of 5 000 cells in 6-well plates previously coated with Collagen I and were incubated for 24h. Then, cells were starved overnight and stimulated with DMEM 10% FCS or DMEM without FCS as control. Cell migration was measured by cell tracking using video-microscopy and Metamorph software (Molecular Device) for 8h.

### Xenograft mouse models

NOD/SCID (non-obese diabetic/severe combined immunodeficient)/γc null mice (NSG) were obtained from Charles River Laboratory (France). HCT15 cells expressing shCTRL or shPTK7 were infected with lentivirus vector expressing LUC2 and 2.10^6^ cells were subcutaneously injected in 9 weeks old NSG mice (weight: 25g). LUC2 expression level was controlled before injection. Tumor development was followed during 8 weeks and calculated using the formula V = 0.52x(LxW^2^). At day 50, tumor resection was performed and tumor weight was measured. Then, development of metastasis was followed by bioluminescence analysis using Photon imageur (Biospace Lab, Paris, France). Upon appearance of metastatic signals or completion of the analysis (day 130), the mice were autopsied and organ luminescence was performed. Image analysis was performed using M3 vision software (Biospace lab). All experiments were performed in agreement with the French Guidelines for animal handling.

### Statistical analysis

Comparison of categorical variable distribution was performed by χ2 or Fisher’s exact test. For continuous variables, Student’s t-test and non-parametric Mann-Whitney U test were used. Follow-up was calculated from diagnosis to the date of last news for patients alive. Metastasis-free survival (MFS) was measured from diagnosis until the first metastatic event or death. For patients without metastatic relapse or death at the time of the last follow-up, censoring was performed at the date of last follow-up. Overall survival (OS) was measured from diagnosis until death or date of last follow-up. Survivals were estimated with the Kaplan–Meier method and compared between groups with the log-rank test. For multivariate analysis, a Cox proportional hazards model was used. The data are reported in accordance with the reporting recommendations for tumor marker prognostic studies (remark) [35]. In order to compare tumor growth curves in preclinical experiments, two-way ANOVA was performed. All statistical tests were two-sided. Statistical analyses were performed either with the R software version 2.15 or Prism software (Graphpad software, San Diego, CA, USA)

## Results

### PTK7 protein is significantly up-regulated in CRC

**Fig 1:**
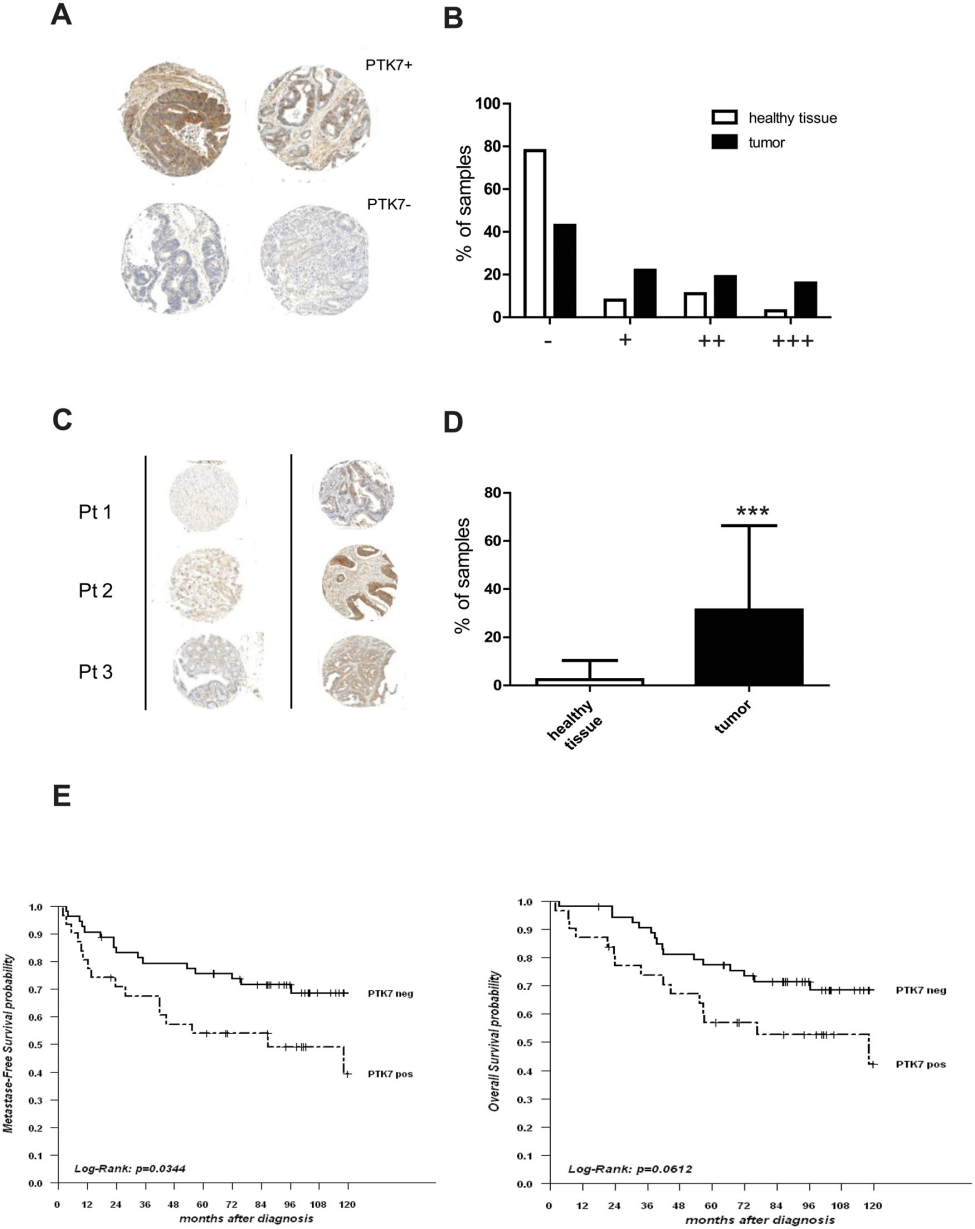
PTK7 is overexpressed in primary CRC and is associated with reduced survival. A- PTK7 expression by immunohistochemistry (IHC) on TMA containing primary CRC tissues: staining of tumor cores showing high (upper panel) and no/low (lower panel) expression in representative cancer tissues (magnification x 400). B- Intensity of PTK7 staining by IHC in primary CRC and matched normal mucosa: staining was classified as 0, 1+, 2+ and 3+ as described in materials and method section. Proportions were compared by Chi-2 test. C-Mean percentage of cells with PTK7 staining in primary CRC and matched normal mucosa. D- Tumor cores of primary CRC showing PTK7 overexpression (as defined by staining of 10% of cells or more, with intensity of 2 or more) and matched normal mucosa (magnification x 400). Percentages were compared using Mann-Whitney U test. *** = p<0.001. E- Kaplan-Meier analysis of the metastasis-free (right) and overall (left) survivals of non-metastatic CRC patients according to PTK7 overexpression on TMA. Survivals were compared using log-rank test.

In order to better understand the role of PTK7 in colorectal tumorigenesis, we examined its protein expression by immunohistochemistry on a TMA containing tumor tissues from 192 primary CRCs (the clinical characteristics of which are shown in S1 Table) and matched normal mucosa. PTK7 immunostaining could be evaluated in 138 CRC (72%) and 142 normal tissues (74%), respectively. We detected PTK7 expression in primary tumors of 78 patients (56%), with an intensity of 2 or 3 in 48 patients (35%) and an essentially cytoplasmic subcellular location (Fig 1A and 1B). By comparison, only 32 patients (22%) had detectable immunostaining in matched normal mucosa, with an intensity of 2 or 3 in 20 patients (14%) (p = 1.91.10^–8^, chi-2 test; Fig 1A and 1B). Moreover, when PTK7 was expressed, the mean percentage number of positive cells was 31% (95%CI: 25–37%) in tumor tissue versus 2% (95%CI: 1.2–3.8%; p = 5.74. 10^–14^, Mann-Whitney U Test) in matched normal mucosa (Fig 1C and 1D). Using a cut-off of at least 10% of positive cells with an intensity of 2 or more (which will be used thereafter as definition of PTK7 overexpression), 47 patients had detectable PTK7 overexpression in tumor tissue (34%) but only 3 in normal mucosa (2%) (Fig 1D). Thus, PTK7 was overexpressed in a significant number of primary CRC, but not or barely in normal tissue.

### PTK7 overexpression is associated with poor survival in non-metastatic CRC

We investigated whether PTK7 overexpression was correlated with main clinical and pathological features. As shown in S2 Table, no significant association was found between PTK7 overexpression and parameters such as age, sex, colon or rectum location, tumor stage, lymph node extension, metastatic stage, lympho-vascular invasion (LVI), differentiation grade and colloid mucous component. Of note, there was a non-significant trend toward a lower expression in tumors with microsatellite instability-high phenotype. We then focused on stage I-III, M0 CRC patients and evaluated the impact of PTK7 overexpression on survival. By univariate analysis, together with lymph node involvement and colloid mucous component, PTK7 overexpression was associated with a significantly reduced MFS (Table 1). The 5-year MFS was 75.7% (95%CI: 65–88.1%) in PTK7-negative tumors versus 54% (95%CI: 38.8–75%) in PTK7-positive tumors (HR = 2.1 [1–4.1]; p = 0.034, log-rank test; Fig 1E). By multivariate analysis, only colloid mucous component retained independent prognostic value for MFS, but PTK7 approached statistical significance (Table 1). Similar trends were observed in terms of overall survival (Fig 1E; Table 1). Thus, PTK7 overexpression in non-metastatic CRC was associated with an adverse outcome. To investigate possible differences between primary and metastatic tumors, we examined additional primary tissues from a small cohort of 7 PTK7-positive liver metastases. In all samples, PTK7 expression was similar in both tissues (S1 Fig)

**Table 1:** Univariate and multivariate analysis of metastasis-free survival (MFS) and overall survival (OS) in non-metastatic colorectal cancer patients.

			Univariate analysis		Multivariate analysis∞	
		variables	Hazard ratio	p-value*	Hazard ratio	p-value**
MFS	Node	N0	1		1	
		N1-2	2.7 [1.5–4.8]	0.0003	2.2 [0.7–6.5]	0.139
	Colloid mucous	No	1		1	
		Yes	2.8 [1.1–7.1]	0.0229	5.2 [1.5–17.7]	0.00757
	PTK7	No	1		1	
		Yes	2.1 [1–4.1]	0.0344	2.3 [0.9–5.5]	0.0603
	LVI	No	1		1	
		Yes	1.6 [0.9–3]	0.1022	1.3 [0.552–3.496]	0.484
OS	Node	N0	1		1	
		N1-2	2.7 [1.5–4.8]	0.0003	2.1 [0.7–6.2]	0.163
	Colloid mucous	No	1		1	
		Yes	2.8 [1.1–7.1]	0.0268	4.3 [1.2–14.3]	0.0173
	PTK7	No	1		1	
		Yes	1.9 [1–3.9]	0.0612	2 [0.8–4.9]	0.13
	LVI	No	1		1	
		Yes	1.6 [0.9–2.9]	0.126	1.3 [0.4–3.3]	0.595

LVI = lymphovascular invasion,

* Log-rank test,

** Cox proportional model,

^∞^ only variables with p-value <0.15 by univariate analysis were put into multivariate analysis

### PTK7 downregulation does not affect cell proliferation and drug-resistance, but it reduces cell migration of human CRC cell lines

**Fig 2:**
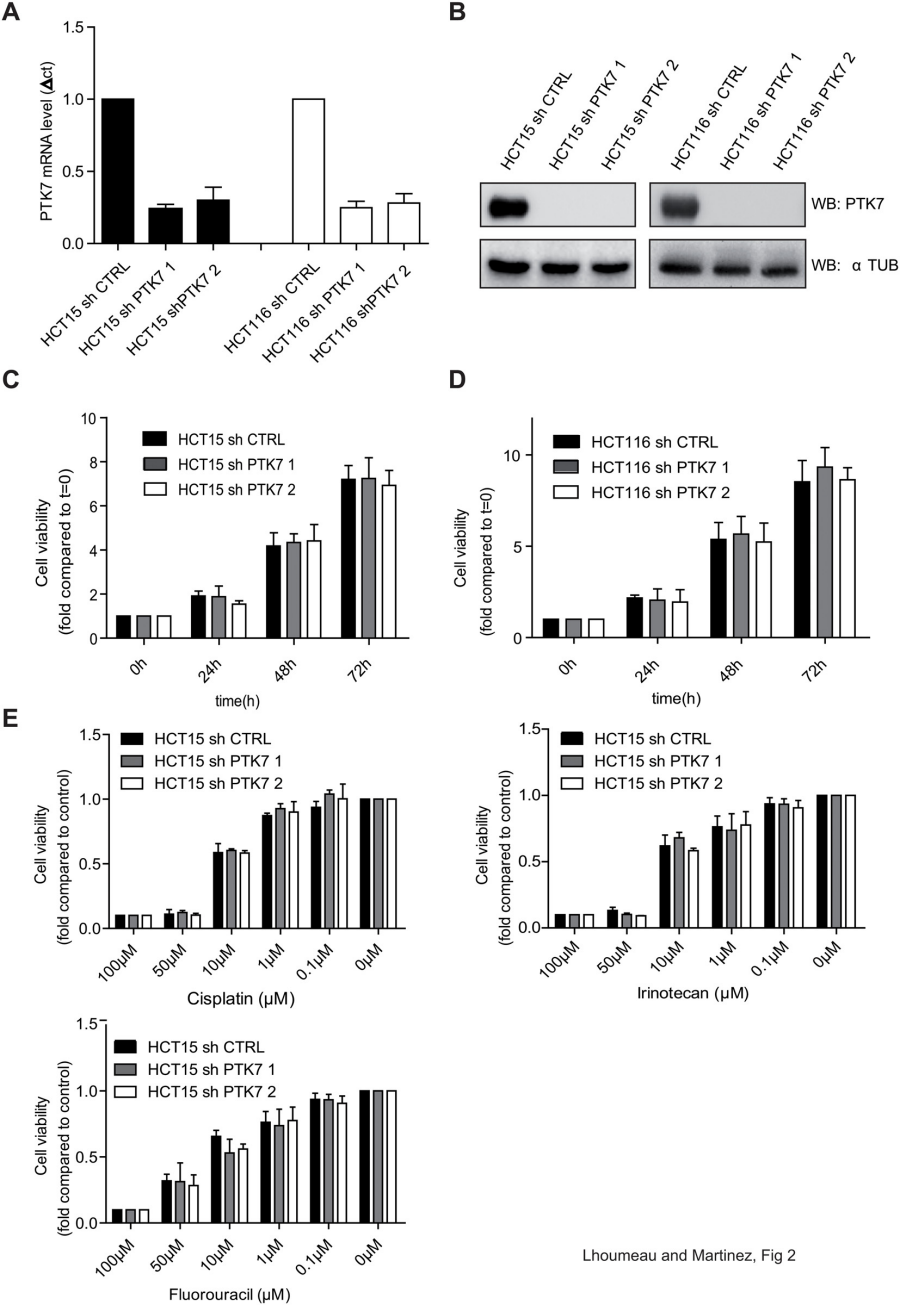
Impact of PTK7 downregulation on cell proliferation and drug resistance. PTK7 expression level in shCTRL- or shPTK7 (1 and 2)-infected HCT15 and HCT116 cell lines was checked by western blot (A) and Q-PCR analysis (B). Cell viability was quantified by cell titer assay for 3 days (C-D). Cell viability of shCTRL- and shPTK7 (1 and 2)-infected HCT15 cells was evaluated by cell titer assay, 72 h after adjunction of cisplatin, Irinotecan, and 5-Fluorouracil (E).

To investigate the biological basis of increased aggressiveness in PTK7 overexpressing CRC, we established a knockdown model of PTK7 in human CRC cell lines HCT15 and HCT116, which express a large amount of endogenous PTK7 (Fig 2A). Cells were infected with lentiviruses expressing a non-targeting shRNA control (shCTRL) or two different shRNA targeting PTK7 (shPTK7-1 and shPTK7-2) and the efficiency of PTK7 knockdown was verified at mRNA and protein levels (Fig 2A and 2B).

We then analyzed the effect of PTK7 on cell proliferation and survival using a cell titer assay. In this proliferation assay, down-regulating the expression of PTK7 in HCT15 and HCT116 cell lines had no effect on proliferative capacity of cancer cells (Fig 2C and 2D). Because PTK7 is involved in WNT pathways, we checked whether its potential impact on proliferation could be dependent on Wnt ligand stimulation [14,18]. Thus, proliferation was further examined in the presence of conditioned media from L cells expressing Wnt3a and Wnt5a, which are known activators of canonical and non-canonical pathways, respectively. However, even under Wnt stimulation, we did not detect any impact of PTK7 expression on CRC cell proliferation (S2 Fig).

We previously found that PTK7 expression enhances drug resistance in acute myeloid leukemia cells [28]. Therefore, to examine its impact on drug resistance in CRC cell lines, we evaluated cell viability of HCT15 cells expressing or not PTK7 after treatment by different doses of cisplatin, irinotecan and 5-FU. As shown in Fig 2E, downregulating PTK7 expression did not confer any supplemental sensitivity of HCT15 to cytotoxics. Similar results were obtained with HCT116 (S3 Fig).

**Fig 3:**
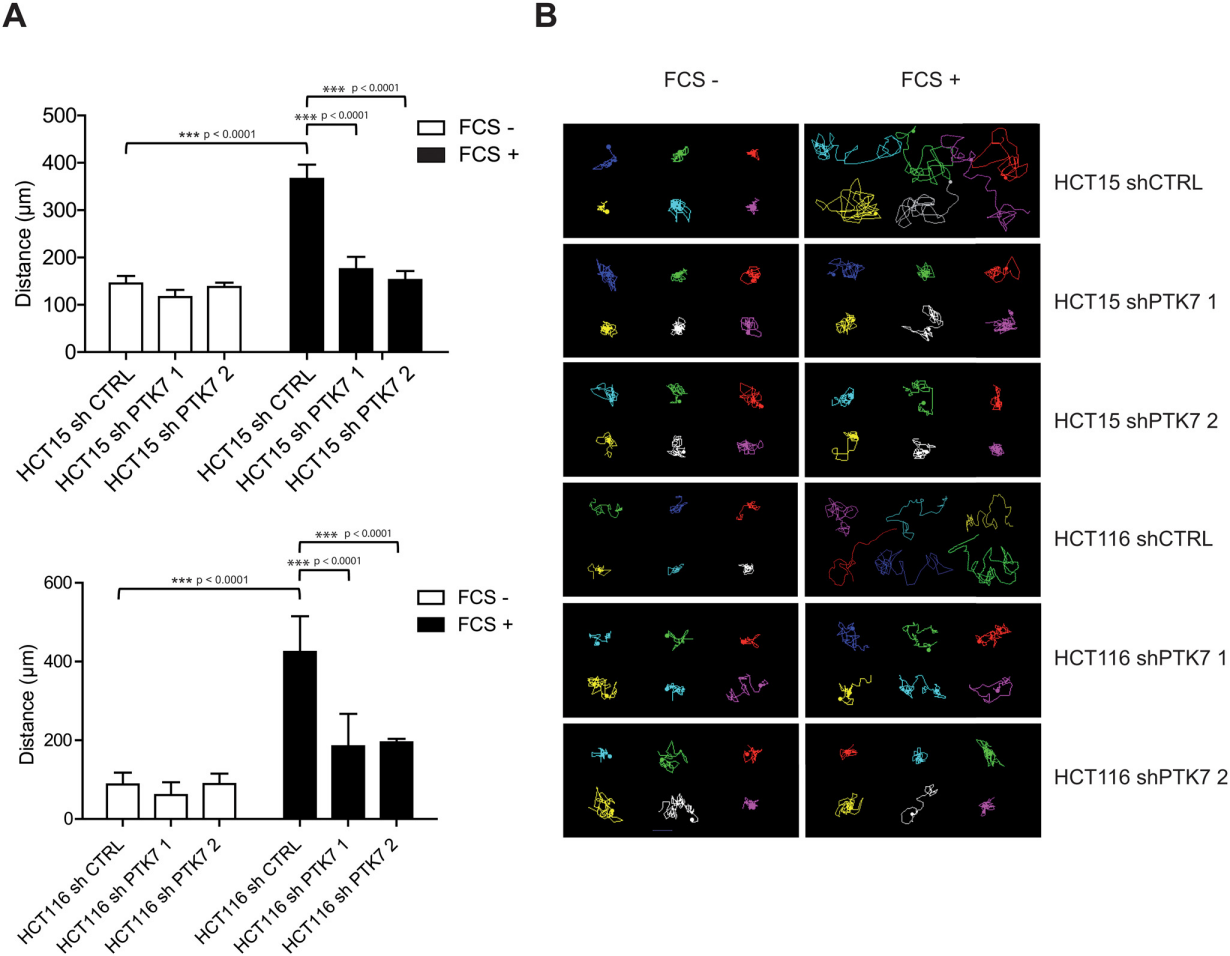
Pro-migratory effect of PTK7 in HCT15 and HCT116 cells. shCTRL- and shPTK7 (1 and 2)-infected HCT15 cells were starved overnight and incubated with or without FCS. Cell migration was measured by cell tracking using video-microscopy and Metamorph software. Distance of migration was calculated for each condition and means were compared using Mann-Whitney U Test (A). Representative migrating courses were shown in (B).

We next investigated the role of PTK7 in cell migration. HCT15 cells were starved for 24h, then stimulated with FCS and cell migration was followed by live-cell tracking. When FCS was added as a stimulating factor, the migration of HCT15 shCTRL cells increased by 2,5 fold. However, in PTK7-knockdown HCT15 cells, FCS-induced migration was abolished. Similar results were found for HCT116 cells (Fig 3A and 3B). Thus, PTK7 expression does not affect either cell proliferation or drug resistance, but it confers pro-migratory capacities to human CRC cell lines.

### PTK7 downregulation reduces tumor growth and metastasis development in xenograft mouse models

**Fig 4:**
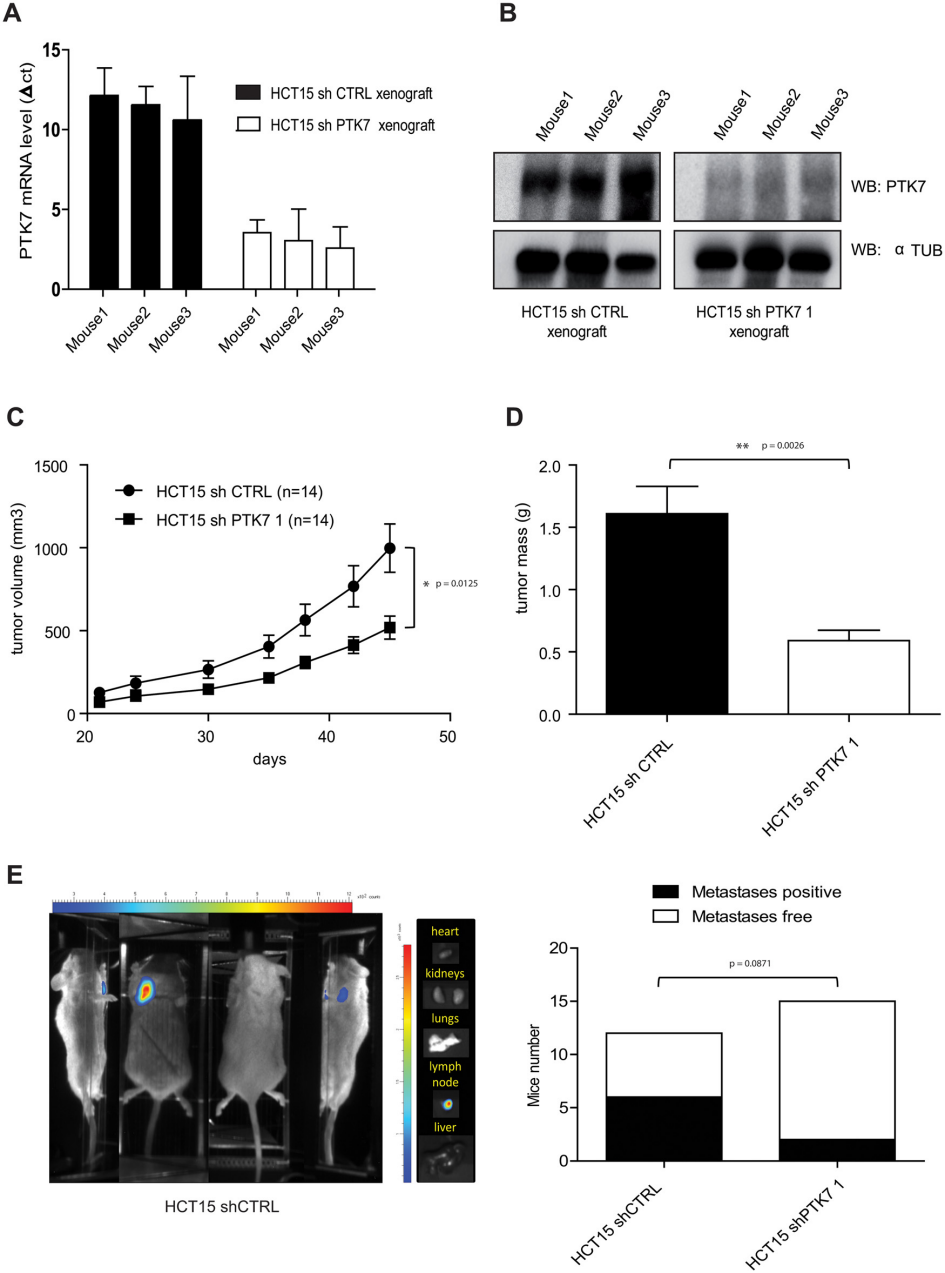
Pro-tumorigenic effect of PTK7 in xenograft model. Expression of PTK7 was checked after tumor resection by Q-PCR (A) and western blot analysis (B). Growth of subcutaneous xenograft of shCTRL- and shPTK7-infected cells HCT15 were examined (C- D). Images from a representative HCT15 shCTRL injected mouse and their organs were shown in left panel. The number of metastase-free or-positive mice in each condition was evaluated and shown in right panel. Proportions were compared using Fischer’s exact test (E).

To examine the tumorigenic activity of PTK7 *in vivo*, we subcutaneously transplanted bioluminescent HCT15 cells (shCTRL or shPTK7) in NSG mice and monitored both tumor growth and metastatic dissemination. As shown in Fig 4A and 4B, the efficiency of PTK7 knockdown was checked in tumors at both mRNA and protein levels. In PTK7 knockdown HCT15 cells, tumor volume was reduced by almost 50% (Fig 4C; p = 0, 0125, two-way ANOVA) as compared to control cells. Similarly, tumor mass was decreased by 2.6 fold in PTK7 knockdown tumors (Fig 4D; p = 0, 0025, Mann-Whitney U test). Thus, PTK7 plays a pro-tumorigenic role *in vivo*. To evaluate whether this pro-tumorigenic impact is due to difference in cell proliferation, we examined the Ki67 status in tumor xenografts, with and without PTK7 knockdown. As shown in S4 Fig, no significant difference was observed between HCT15 shCTRL and HCT15 shPTK7 xenografts in terms of Ki67 staining.

With regards to the clinical impact of PTK7 overexpression on MFS and its pro-migratory effect *in vitro*, we examined whether PTK7 could enhance metastasis in xenografted mice model. After resection of HCT15-derived tumors, metastatic development was monitored *in vivo* using a luciferase reporter. There was a non-significant trend for less metastatic occurrence in mice injected with PTK7-knockdown HCT15 cells, compared to shCTRL-HCT15 injected mice (2 metastatic events in 15 mice versus 6 metastatic events in 12 mice, respectively; p = 0.0871, Fischer’s exact test) (Fig 4E).

To further investigate a potential pro-metastatic effect of PTK7, we built a cellular model of PTK7 overexpression in PTK7-negative cancer cells and examined its impact on metastasis development *in vivo*. In the absence of any available PTK7-negative human CRC cells, we used a B16F10 melanoma cell line, a PTK7-negative cell with well documented ability to produce lung metastases in xenograft models [36], as a model. We transfected B16F10 cells with PTK7 expression or empty vector, and check that PTK7 was stably expressed at the plasma membrane by FACS analysis (S5A and S5B Fig). B16F10 cells were then injected intravenously and lung metastasis development was analyzed. We found that overexpression of PTK7 significantly enhanced the metastatic development (p = 0.0024) (S5C and S5D Fig). Looking at the size of metastases, we found that PTK7 overexpression led to an increased number of small and large metastases (p = 0.0019 and 0.0080, respectively; S5E Fig). However, the proportion of small metastases was higher in PTK7 overexpressing cells, suggesting a predominant effect on cell invasion and adhesion, rather than on cell proliferation. Thus, PTK7 overexpression enhances the metastatic phenotype *in vivo*.

## Discussion

While aberrant mRNA expression of PTK7 was originally detected in CRC [13], no data were available about its protein expression in clinically annotated CRC samples. Using immunohistochemistry, we have confirmed its protein overexpression in a relevant fraction of CRC cases (around one-third of patients), compared to matched healthy mucosae where it was not or barely detectable. In this series, expression was not found to be associated with any specific clinical or pathological features, including age, tumor size, lymph node involvement, stage, LVI, colloid mucous component, and differentiation grade. However, in non-metastatic CRC, PTK7 overexpression was associated with a significant reduced MFS. This poor-prognosis impact in term of metastatic potential was found to approach statistical significance in a multivariate analysis, whereas lymph node involvement was not retained. Regarding to OS, similar trends were observed but without reaching statistical significance, most likely due to the limited sample size. Of note, no significant link between PTK7 overexpression and metastatic stage on diagnosis was found, raising the intriguing hypothesis that PTK7 expression might be lost once metastases are established. However, we examined PTK7 expression in paired primary tissues from PTK7-positive metastatic tissues and found that it remained conserved at primary site in all cases.

Therefore, PTK7 may indicate a higher probability of metastatic event, independently of already known prognostic parameters, including the current major criteria for adjuvant chemotherapy, i.e. lymph node involvement. Thus, if validated on further independent dataset, PTK7 could be used as a biomarker helpful to distinguish node-negative patients that may require systemic adjuvant therapy from and node-positive patients that could be spared from chemotherapy side effects. We recently described a similar poor-prognosis impact for PTK7 expression in AML patients, in whom it was associated with a higher relapse rate and reduced leukemia-free survival and OS [28]. Data reported in the literature in some other malignancies, including liposarcoma, oesophageal squamous cell carcinoma and invasive cholangiocarcinoma [22,25,27], are also consistent with a similar adverse impact of PTK7 expression on clinical outcome. In breast cancer, PTK7 overexpression was found to be associated with the highly aggressive triple-negative subtype and its high expression in lymph node was associated with reduced disease-free survival [24]. However, in another series of triple-negative breast cancer patients, a shorter disease-free survival was only seen in patients receiving anthracylines-based chemotherapy [23]. Moreover, PTK7 expression was associated with a more favorable outcome in gastric or lung cancer [21,37]. Thus, PTK7 prognostic impact could be dependent on tumor types and/or administered treatments.

To investigate the biological basis for the increased metastatic ability that we observed in the clinical setting, we examined the impact of PTK7 downregulation in human CRC cell lines using specific shRNA. Importantly, we found that PTK7 silencing induced a significant reduced capacity to migrate in a live-cell tracking assay. Consistently, xenotransplantation of human PTK7-depleted CRC cells resulted in significant reduced tumor growth and a trend toward less metastatic events, compared to control cells. Finally, overexpression of PTK7 in a PTK7-negative model of melanoma was associated with an increased number of metastases. This was consistent with several lines of preclinical data, including our own with cultured AML cells in which PTK7 ectopic expression increased spontaneous and serum-induced migration, whereas PTK7 depletion decreased cell migration [28]. A similar pro-migratory phenotype was also shown in various epithelial models [22,24,25], including colon cancer [38]. Obviously, this phenotype could rely upon the demonstrated function of PTK7 in regulating PCP, which a central role in a large number of cell processes related to cell adhesion, cell motility and invasion [39]. In addition, the impact on the metastatic phenotype may also result from the role of PTK7 in endothelial cell tube formation and migration during the angiogenic process [40].

Surprisingly, in our *in vitro* experiments, PTK7 depletion was not found to significantly affect cell proliferation, as opposed to results generated by Meng et al [41], using the same HCT116 cell line. Reasons for such different results are not clear, but in our xenograft experiments with HCT15 cells, tumor growth was significantly decreased in PTK7-depleted cells, whereas the Ki67 status did not shown any significant difference, consistently with *in vitro* results (S4 Fig). Thus, in our model, the impaired tumor growth *in vivo* in PTK7-depleted cells could rather result from an increase in apoptosis. The impact of PTK7 expression on cell survival was not detected *in vitro* but this could result from a specific requirement for a yet to be determined extracellular signal, present in tumor stromal environment but not in cell culture conditions. In the Meng study, anti-PTK7 siRNA induced a reduction in cell viability, cell proliferation and an increase in caspase-10-dependent apoptosis. A similar positive correlation between PTK7 expression and increased cell proliferation and/or survival was also shown in other models. In AML cells, survival and resistance to apoptosis induced by anthracyclines were modulated by the level of PTK7 expression, being increased in PTK7-expressing cells and decreased in PTK7-depleted cells, whereas proliferation was not affected [28]. In oesophageal squamous cell cancer, PTK7 knockdown decreased cell proliferation, presumably due to a decrease in phosphorylation levels of Akt, Erk, JNK, p38 MAPK, and FAK [22]. In cholangiocarcinoma cells, PTK7 depletion affected the level of cell-cycle related proteins, increasing Cdk2, Cdk4, Cdk6, and cyclin D1 and decreasing p16, p21, and p27, whereas it increased mitochondrial-dependent apoptosis. Of note, Na et al [38] have shown that specific biological activity of PTK7 may actually be dependent on the generation of distinct extracellular or cytosolic fragments from the full length protein by proteolytic events involving metalloprotease ADAM17 and γ-secretase activities. Such events may require a specific cell context, potentially explaining inconsistencies between cell lines or tumor types, when examining the impact of PTK7 modulation. Consistent with this observation, a recent paper [42] showed that full-length membranous PTK7 and different mutant forms at cleavage sites for membrane type-1 matrix metalloproteinase differentially regulated cell motility, invadopodia, lamellipodia protrusions, invasion and metastases. Moreover, both PTK7 overexpression and silencing were able to inhibit pro-metastatic and invasive phenotype. In addition, both full-length and proteolytic PTK7 fragments were detected in human colon cancer tissues and it was hypothesized that the ratio of proteolytic fragments to full-length forms, rather than PTK7 total expression, could be biomarker in cancer. Of note, the antibodies used in this study were able to detect both full length membranous PTK7 and its soluble form, without distinction (S6 Fig). Notwithstanding this debate, anti-tumor effects observed when depleting PTK7 expression, as exemplified in our study as in others, indicate that it may represent a valuable target for different cancer therapeutics including CRC. Preclinical evidence for reversal of PTK7 pro-tumorigenic effects by adding soluble PTK7 extracellular domain or by expression of a mutant form with deletion of kinase domain in AML cells [28] supports this therapeutic potential.

Targeted therapies have been largely introduced in management of CRC, including anti-EGFR monoclonal antibodies or anti-angiogenic agents that have provided significant survival improvements in metastatic patients. However, all have failed to significantly affect early-stage disease and novel therapeutic targets involved in crucial cellular functions, such as cell migration and metastatic development, are urgently needed for this disease. According to our results, including its overexpression in a significant percentage of patients that can be easily detected by IHC, its demonstrated prognostic impact in terms of MFS, and the observed anti-metastatic properties by specific shRNA depletion in preclinical models, PTK7 may represent a promising candidate to be further examined.

## Supporting Information

S1 Fig PTK7 IHC on paired primary CRC and liver metastases. PTK7 expression was examined by immunohistochemistry (IHC) as described in materials and method section, on 7 PTK7-positive liver metastases with paired primary CRC. Two representative cases are shown: a patient with metastases occurring one year after diagnosis (A) and a patient with synchronous liver metastases (B) (X10).
(PDF)

S2 Fig Proliferation of HCT15 cells under Wnt-ligands stimulation. shCTRL- and shPTK7 (1 and 2)-HCT15 cells were grown in control media (A) or in Wnt3a- (B) and Wnt5a- (C) conditioned medium and viable cells were measured using cell titer assay after 1, 2, 3 or 4 days of culture.
(EPS)

S3 Fig Drug resistance of HCT116 cells. Cell viability of shCTRL- and shPTK7 (1 and 2)- HCT116 cells was evaluated by cell titer assay, 72 h after adjunction of Irinotecan (A), cisplatin (B), and 5-Fluorouracil(C).
(EPS)

S4 Fig Cell proliferation in tumor xenograft models. Ki67 was evaluated by IHC in paraffin‐embedded tissue from subcutaneous xenograft of shCTRL and shPTK7-infected cells HCT15 (5X/10X, counterstaining with hematoxylin).
(PDF)

S5 Fig B16F10 *in vivo* metastasis assay. Overexpression of PTK7 was checked by western blot (A) using BAF3 cells as control and by immunofluorescence showing correct expression at the cell membrane (B). Representative macroscopic pictures of the lungs of B16F10 transfected with empty-vector(C left panel) and with full length of PTK7 (C right panel). (D) Quantification of total metastasis in B16F10-control and B16F10-PTK7 injected mice. (E) Evaluation of tumors size and (F) proportion of small tumors in B16F10-control and B16F10-PTK7 injected mice. Results are representative of two independent experiments done with 10 mice in each group. Mean number and proportion of metastases in each condition were compared using Mann-Whitney U test and Fischer’s exact test, respectively. ** = p<0.01; * = p<0.05.
(EPS)

S6 Fig Immunodetection of both full-length and soluble forms. After FLAG or FC pull down on cell lysates expressing empty vector and PTK7-FLAG or cell supernatant containing sPTK7-FC, Western Blot were performed using rat monoclonal anti-PTK7 generated in the laboratory or anti-human PTK7/CCK-4 affinity-purified polyclonal antibody obtained from R&D Systems. Tubulin and Ponceau S are shown as loading control.
(EPS)

S1 Materials and Methods (DOC)

S1 Table Patient population. (EPS)

S2 Table Correlations between PTK7 expression and clinico-pathological features. (EPS)
