# Supplementary figures and images for: Overexpression of the Promigratory and Prometastatic PTK7 Receptor Is Associated with an Adverse Clinical Outcome in Colorectal Cancer
Source: PLoS One. 2015 May 11;10(5):e0123768. doi: 10.1371/journal.pone.0123768 (PMC4427440; doi:10.1371/journal.pone.0123768)

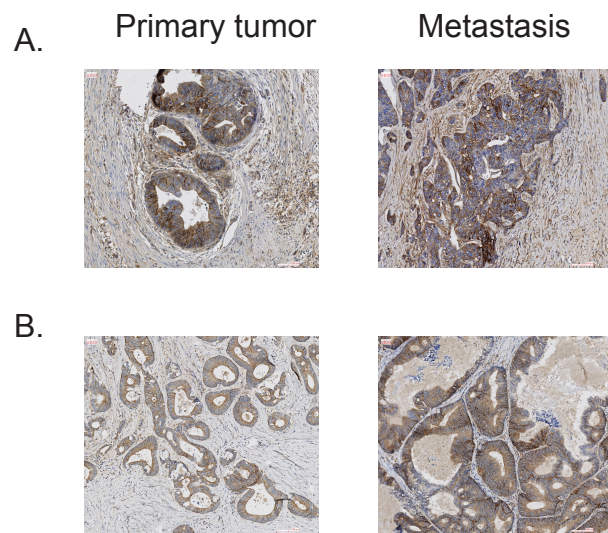

Lhoumeau and Martinez. Figure S1

Supplement: S1 Fig — PTK7 expression was examined by immunohistochemistry (IHC) as described in materials and method section, on 7 PTK7-positive liver metastases with paired primary CRC. Two representative cases are shown: a patient with metastases occurring one year after diagnosis (A) and a patient with synchronous liver metastases (B) (X10). (PDF) [file pone.0123768.s001.pdf]

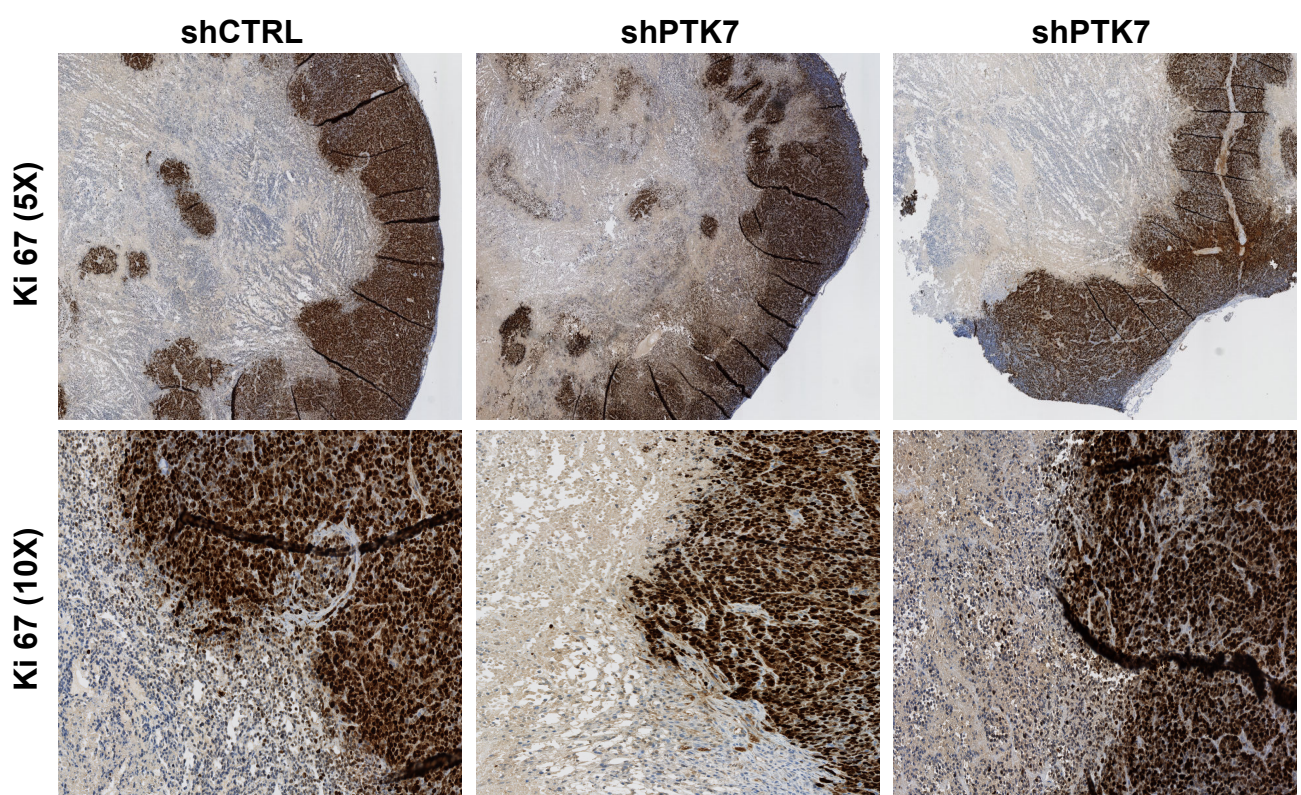

Lhoumeau and Martinez, Fig S4

Supplement: S4 Fig — Ki67 was evaluated by IHC in paraffin‐embedded tissue from subcutaneous xenograft of shCTRL and shPTK7-infected cells HCT15 (5X/10X, counterstaining with hematoxylin). (PDF) [file pone.0123768.s004.pdf]
