# Supplementary material for: Overexpression of the Promigratory and Prometastatic PTK7 Receptor Is Associated with an Adverse Clinical Outcome in Colorectal Cancer
Source: PLoS One. 2015 May 11;10(5):e0123768. doi: 10.1371/journal.pone.0123768 (PMC4427440; doi:10.1371/journal.pone.0123768)
Supplement: S1 Materials and Methods — (DOC) [file pone.0123768.s007.doc]

**Supplementary material and methods**

***shRNA sequences***

***L-Cells and Wnt5 ant Wnt3 production***

L-Cells were obtained from ATCC and were maintained in Dulbecco’s Eagle Modified Medium supplemented with 10% FBS. Cells were split 1:10 in 10 mL culture medium in 10 cm petri dishes and grown for 4 days approximately to reach confluence. After recovering a first batch of medium, 10 ml of fresh culture were added and culture was prolonged for another 3 days. A second batch of medium was then recovered and mixed with the first at a ratio 1:1.

***B16F10 in vivo metastasis assay***

B16F10 cells were obtained from ATCC and were maintained in Dulbecco’s Eagle Modified Medium supplemented with 10% FBS. Stable B16F10-control cells and B16F10-PTK7 were generated using electroporation (Amaxa nucleofector, Lonza, Switzerland) with a mock pcDNA3 vector or with the pcDNA3-human PTK7-Flag construct already described by Shin et al(1). B16F10 cells (2.10^5^ cells/mouse) were injected i.v. in C57BL/6 mice. Mice were sacrificed 14 days after and lung removed in PFA 4%. Number and size of metastasis were counted: tumors smaller than 0.5mm diameter (i.e.<0.2mm^2^) are defined as small tumors. For immunofluorescence, we used rabbit polyclonal anti-mouse PTK7 (39) and secondary AF488 donkey anti rabbit (Jackson Immunoreasearch-Europe, UK).
